# The omitted symptoms challenge the diagnosis of right atrial myxoma: a case report

**DOI:** 10.1186/s12872-020-01413-4

**Published:** 2020-03-25

**Authors:** Shulun Ma, Qian Xu, Ruizheng Shi, Xin Zhang, Xuliang Chen

**Affiliations:** 1 Department of Cardiovascular Surgery, Xiangya Hospital, Central South University, Xiangya Rd 87, Changsha, 410008 China; 2 Department of Cardiovascular Medicine, Xiangya Hospital, Central South University, Changsha, China

**Keywords:** Myxoma, Diagnosis, Symptoms

## Abstract

**Background:**

Cardiac myxoma (CM) is the most common type of primary cardiac tumors. The prevalence of primary cardiac tumors is 0.0017–0.28% in various autopsy studies. The clinical symptoms of CM which includes embolism, intracardiac obstruction, general or constitutional manifestations and infected myxoma are largely depended on the size, growing speed, location and pedicle length of the tumor. The following case reported a missed diagnostic case of a right atrial myxoma firstly presented digestive, systemic symptoms and immunologic disorder, leading to emergent tricuspid valves obstruction situation.

**Case presentation:**

We reported a critical case of a 51-year-old female with CM was firstly admitted to the gastroenterology clinical department because of poor appetite, marked fatigability and weight loss for 2 months. The physician diagnosed her as chronic gastritis and treated her with some symptomatic treatment such as ilaprazole and magnesium aluminum carbonate. After months without definitive diagnosis, this right atrial myxoma grew into right ventricle and obstructed the tricuspid valves, causing her dyspnea, sweating, dizziness, feeling of impending death when she was sleeping. Transthoracic echocardiogram revealed a 6.1 × 4.2 × 3.7 cm^2^ mass adjacent to tricuspid valves. The patient underwent surgical excision and pathology revealed a primary cardiac myxoma.

**Conclusion:**

This case reported a critical result of missed diagnosis of right atrial myxoma and showed its systematic symptoms and immunologic disorder, highlighting the importance of systematic examinations on patients. Furthermore, it appeals early diagnosis of CM and consideration of drug targets to suppress CM development.

## Background

Cardiac myxoma (CM) is the most common type of primary cardiac tumors. The prevalence of primary cardiac tumors is 0.0017–0.28% in various autopsy studies [1]. The clinical symptoms of CM which includes embolism, intracardiac obstruction, general or constitutional manifestations and infected myxoma are largely depended on the size, growing speed, location and pedicle length of the tumor [2]. Thus, the various manifestations challenge the diagnosis of CM and even lead to critical results. Here, we reported a missed diagnostic case of a right atrial myxoma firstly presented digestive, systemic symptoms and immunologic disorder, which caused emergent tricuspid valves obstruction situation.

## Case presentation

A 51-year-old female patient without special family history was admitted to the gastroenterology clinical department because of poor appetite, marked fatigability and weight loss for 2 months. Her physical examinations revealed no evidence of abnormalities: no edema or superficial varicosities. Cardiac exam revealed normal heart rate and rhythm without extra sounds or murmurs. No further cardiac examination was done based on her chief complaint and normal physical examinations. Her abdomen was soft. Superficial and deep palpation didn’t find organomegaly or masses. The clinical examinations revealed normal results of blood routine, urine routine, stool routine, liver function and gastroscopic, except for slightly high globulin (39.3 g/L) and low albumin (28 g/L). Then, quantitative measurements of plasma immunoglobulin and coagulation were performed. The results showed plasma IgE was 413 IU/mL and IgG was 17.4 g/L. PTA (prothrombin activity) decreased to 56.8% while PT (prothrombin time) and fibrinogen increased to 14.5 s and 4.83 g/L respectively. In order to exclude any hematological system diseases, bone marrow puncture was performed and result revealed elevated level of plasmocytes (8%). The physician diagnosed her as chronic gastritis and treated her with some symptomatic treatment such as ilaprazole and magnesium aluminum carbonate. Finally, the patient felt better and left.

**Fig. 1:**
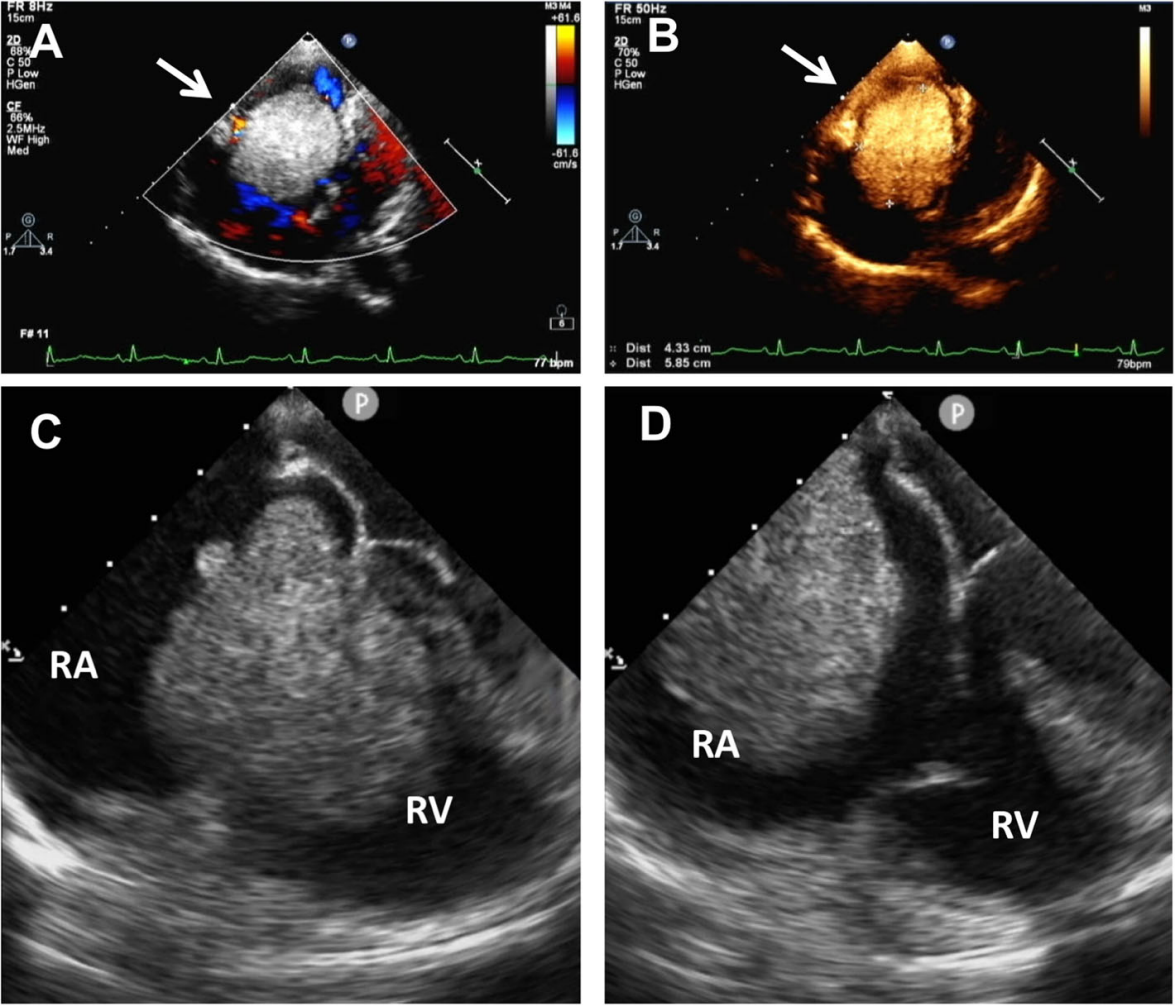
Transthoracic echocardiography showed a giant mass (white arrow) in the right atrium (**a**-**b**). Transesophageal echocardiogram clearly showed the mass obstructed into the tricuspid valves and retracted to the right atrium afterwards (**c**-**d**). RA: right atrium; RV: right ventricle


One month later, the woman suddenly felt dyspnea, sweating, dizziness and feeling of impending death when she was sleeping. The symptoms slightly relieved when she slept in left lateral position or sat up. She was referred to our hospital for emergency call and a series of clinical examinations were performed. Transthoracic echocardiogram revealed a mobile 6.1 × 4.2 × 3.7 cm^2^ mass with irregular borders and hyperechogenicity in the right heart, adjacent to the tricuspid valves (Fig. 1a-b). Half of the mass located in the atrium and the other half was in the ventricle. Obstructed by the mass, the tricuspid valves are unable to close which correlated with her tricuspid valves area systolic murmur. The transesophageal echocardiogram clearly showed the mass obstructed into the tricuspid valves (Fig. 1c-d, Video 1). The electrocardiogram demonstrated incomplete right bundle branch block, right atrium enlargement, II, III, avF ST depression and right deviation of electrocardio axis. Computed tomography result indicated that right atrium dilated. No pleural effusion and enlarged mediastinal lymph nodes were found. Magnetic resonance imaging was not performed because the patient was incapable of lying down.

**Additional file 1.**



**Fig. 2:**
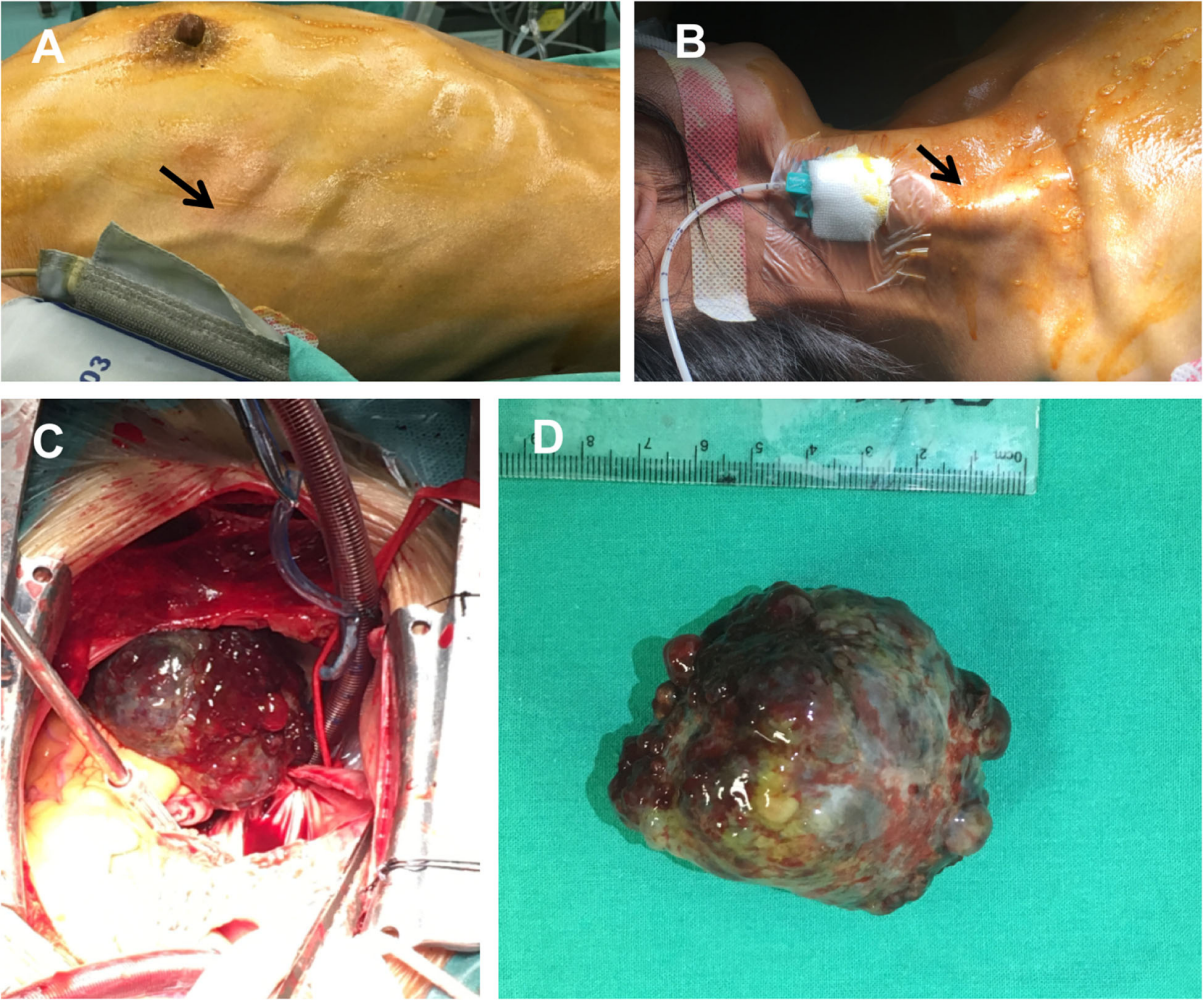
The distention of chest wall (**a**) and jugular vessels (**b**) (black arrow) on patient. During the surgery, we found a giant right atrial myxoma measuring 65 mm × 45 mm × 35 mm was located adjacent to tricuspid valves (**c**, **d**)

**Fig. 3:**
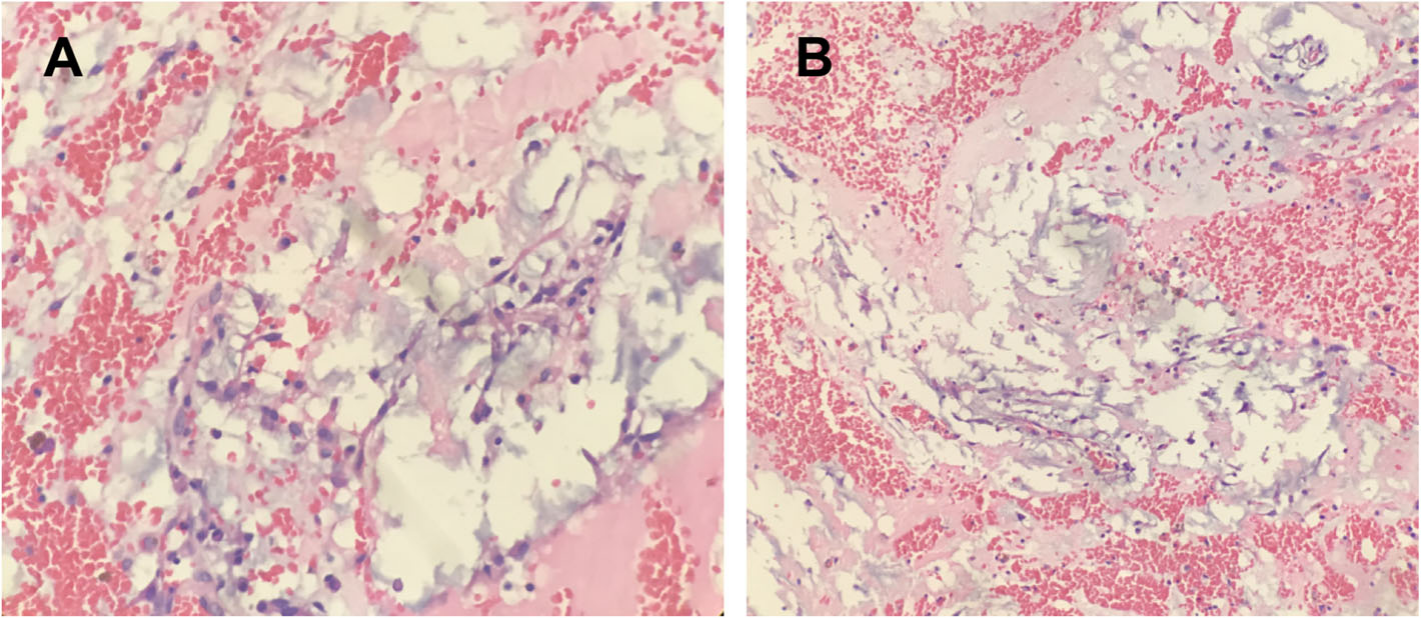
Pathological examination showed star-shaped tumor cell with halos around them. Large scale of hemorrhage occurred in the myxoma (**a**, **b**). Magnification is 400× for 2E and 200× for 2F

At the time of perioperative period, the patient with apparent varicose chest vein had to maintain the left lateral position because of the mass obstruction (Fig. 2a-b). Even before the extracorporeal circulation, her systolic pressure had dropped maximal to 45 mmHg for several times due to the obstruction of the tumor into the tricuspid valves. Extrusion of the tumor by hands immediately was effective and blood pressure returned to normal. At the end, the mass was removed and the patient’s symptoms relieved (Fig. 2c-d). The pathological examination verified that the mass was a primary cardiac myxoma (Fig. 3a-b). She was discharged home 7 days after the operation and is currently asymptomatic and doing well after 12 months of follow-up.

## Discussion and conclusion

Cardiac myxoma (CM) is the most common type of primary cardiac tumors. About 75% of myxomas generate from the left atrium, 10–20% in the right atrium, and rarely in the ventricles [1, 3]. In this case, the patient firstly presented digestive and systemic manifestations including weight loss, poor appetite and marked fatigability. It was considered due to autocrine cytokine production such as IL-6 and IL-8 [4]. Her auxiliary examination revealed a high immunoglobulin and increased plasmocytes in bone marrow. This might attribute to the hemorrhage or denaturation of the tumor which led to a series of immunological reactions. Other findings such as chronic hemolytic anemia, thrombocytopenia and polycythemia can also be seen in some cases [5, 6]. These may lead to misdiagnosis of infective endocarditis, vasculitis, rheumatoid arthritis and other systemic diseases [7, 8]. When tumor size grows, it can present symptoms of intracardiac obstruction such as left and right heart failure [9, 10]. The critical right CM in our case showed a severe tricuspid obstruction and lead to dyspnea, orthopnea and systemic congestion. Under this situation, surgery is the only effective treatment.

At present, 34 proteins markers are found to be involved in the histogenesis and development of CM [6]. And some potential drug targets such as CCR2, TGFβ, MUC1, FGFR, EGFR, GATA4, HAND1, MYC, FOS and MMP9 were confirmed to be key nodes. Since CM development [6, 11] can be suppressed by blocking these targets, it is necessary to diagnose CM early in order to provide more treatment options and reduce mortality risk. This case reported a critical result of missed diagnosis of right atrial myxoma and showed its systematic symptoms and immunologic disorder, highlighting the importance of systematic examinations for early diagnosis on patients.

## Abbreviations

CM: Cardiac myxoma
PT: Prothrombin time
PTA: Prothrombin activity
